# Pharmacological preeclampsia prophylaxis: Are high-risk patients being left behind? A cross-sectional study at a Brazilian hospital

**DOI:** 10.61622/rbgo/2026rbgo68

**Published:** 2026-10-13

**Authors:** Heloísa Freitas Fernandes Marques, Ingrid Lourenço, Caio Ribeiro Vieira Leal, Mário Dias Corrêa

**Affiliations:** 1 Universidade Federal de Minas Gerais Hospital das Clínicas Department of Obstetrics and Gynecology Belo Horizonte MG Brazil Department of Obstetrics and Gynecology, Hospital das Clínicas, Universidade Federal de Minas Gerais, Belo Horizonte, MG, Brazil; 2 Universidade Federal de Minas Gerais Faculdade de Medicina Department of Obstetrics and Gynecology Belo Horizonte MG Brazil Department of Obstetrics and Gynecology, Faculdade de Medicina, Universidade Federal de Minas Gerais, Belo Horizonte, MG, Brazil

**Keywords:** Preeclampsia, Prophylaxis, Aspirin, Calcium, Prenatal care, Prenatal diagnosis, Postpartum period, Risk stratification

## Abstract

**Objective:**

To evaluate low-dose aspirin and calcium prescription rates among high-risk preeclampsia (PE) patients at a Brazilian tertiary hospital and identify predictors of prophylaxis failure.

**Methods:**

Cross-sectional study conducted at a Brazilian maternity hospital, in 2025, under guidelines recommending calcium for PE prevention. We included 100 high-risk pregnant/postpartum women who initiated prenatal care before the 12th gestational week. Prescription was adequate if initiated before the 16th week. Separate multivariable logistic regressions identified independent predictors of adequacy (p<0.05).

**Results:**

Among 100 women, 87% had at least one major risk factor; 13% had only multiple moderate factors. Adequate prescription before the 16th week was 54% for aspirin and 77% for calcium. Patients with only moderate factors had the lowest rates (23.1%). In multivariable analyses, chronic hypertension was independently associated with adequate prescription for aspirin (AOR 3.07; 95% CI 1.21–7.80; p=0.018) and calcium (AOR 4.11; 95% CI 1.26–13.46; p=0.019). Initial care at our center predicted adequate aspirin prescription (AOR 5.21; 95% CI 1.95–13.91; p<0.001) and showed a trend for calcium (AOR 3.24; 95% CI 0.98–10.79; p=0.054). Intervention omissions overlapped: 95.7% of patients without calcium also lacked aspirin.

**Conclusion:**

Pharmacological PE prophylaxis is driven by high-visibility clinical comorbidities and the structural setting of initial care. While prominent maternal risks trigger provider adherence, moderate risk factors face structural bottlenecks at lower-level facilities, rendering optimal aspirin initiation dependent on tertiary insertion. Although recent guidelines withdrew routine calcium recommendations, this study highlights that real-world adherence within the referral network was sub-optimal due to early prenatal screening bottlenecks.

## Introduction

Hypertensive disorders of pregnancy (HDP), including preeclampsia (PE), gestational hypertension and chronic hypertension (CH), are among the most frequent complications affecting gestation. Among these, PE stands out due to its potential severity and significant association with adverse maternal and perinatal outcomes.^(1)^ Globally, the prevalence of PE is estimated at 4.6%, varying significantly according to geographic region and socioeconomic development.^(2)^ These rates can be as high as 14.5% in Tanzania and 13.0% in Peru, illustrating the burden in sub-Saharan Africa and parts of Latin America.^(2)^ Conversely, prevalence remains notably lower in high-income countries, as exemplified by Poland, where the rate is approximately 1.4%.^(2)^ In Brazil, PE is the leading cause of maternal mortality, where its pooled prevalence is around 6.7%.^(3)^

Given the severe adverse outcomes associated with PE, significant efforts have been directed toward mitigating its clinical impact. Specifically, preventive interventions, notably low-dose aspirin use in high-risk patients, have demonstrated a substantial reduction in the incidence of PE and other related adverse outcomes, such as perinatal mortality, preterm birth and intrauterine growth restriction.^(4,5)^ However, evidence suggests that these benefits are limited to high-risk individuals, with no significant impact observed in the general population.^(5)^ Yet not completely understood, the underlying mechanisms through which low-dose aspirin reduces PE rates are related to the improvement of the placentation process through early intervention, the inhibition of platelet aggregation to prevent placental infarcts and the promotion of anti-inflammatory effects with endothelial stabilization.^(6)^

Calcium supplementation is another pharmacological prophylaxis for PE that has recently been the subject of debate. It was previously established that both low- and high-dose calcium supplementation were effective in preventing PE among pregnant individuals with low dietary calcium intake.^(7,8)^ However, a groundbreaking meta-analysis of 10 randomized controlled trials involving over 37,000 patients, published in 2025, challenged this consensus by showing that calcium supplementation may result in little to no difference in PE incidence compared to placebo.^(9)^

Currently, the Brazilian Ministry of Health, the Brazilian Network for Studies on Hypertension in Pregnancy (RBEHG), the country's leading expert consensus, and the Brazilian Federation of Gynecology and Obstetrics Associations (FEBRASGO) recommend low-dose aspirin (100 mg/day) for all high-risk individuals.^(10)^ In light of this shifting scientific evidence, in 2026, both RBEHG and FEBRASGO issued an official position statement modifying their consensus guidelines, now explicitly stating that calcium is no longer recommended for the prevention of PE in Brazil.^(11)^ Aspirin should be initiated at the 12th week of gestation, ideally no later than the 16th week. According to the RBEHG, high risk is defined by the presence of at least one major risk factor or two moderate risk factors, as detailed in chart 1.

**Chart 1:** Clinical factors used in PE prediction, according to RBEHG

**Major risks**	
	History of preeclampsia
	Multiple gestation
	Obesity
	Chronic hypertension
	Type 1 or type 2 diabetes
	Chronic kidney disease
	Autoimmune diseases: lupus, antiphospholipid syndrome
	Pregnancy resulting from assisted reproduction
**Moderate risks**	
	Nulliparity
	Family history of preeclampsia (mother, daughter or sister)
	Advanced maternal age (over 35 years old)
	Interpregnancy interval greater than 10 years
	Low socioeconomic status
	Ethnicity: black or "*parda*" (brown)
	Previous pregnancy with adverse outcome related to placental dysfunction: placental abruption, fetal growth restriction, preterm labor or stillbirth

Therefore, this study aims to evaluate the prescription rates of low-dose aspirin and calcium among patients with identifiable risk factors for PE at a Brazilian tertiary hospital. Specifically, we sought to identify patients who met the criteria for prophylaxis and to determine the clinical and systemic factors associated with the adequacy of these prescriptions. By analyzing the influence of maternal comorbidities and the setting of prenatal care, this study aims to expose the gaps between current clinical guidelines and bedside practice.

## Methods

A cross-sectional observational study was conducted at a tertiary referral maternity hospital in Brazil, between July 2025 and January 2026. A convenience sample of 100 high-risk pregnant or postpartum women who initiated prenatal care before the 12th gestational week was established by sequentially recruiting all eligible patients managed within this defined timeframe. As a specialized center for high-risk pregnancies, the institution serves as a reference hub for patients requiring specialized prenatal care. The study population consisted of women aged 18 years or older, admitted to the maternity ward or followed at the high-risk outpatient clinic, regardless of whether their prenatal care was conducted within or outside this facility. To be included, participants, recruited after the 20th week of gestation or during the postpartum period, had to present at least one major or two moderate risk factors for PE, according to the criteria established by the RBEHG,^(10)^ and must have initiated prenatal care before the 12th week of gestation, with gestational age confirmed by the last menstrual period and validated by ultrasound, or by the first ultrasound alone.

The primary objective was to retrospectively assess the rates of aspirin and calcium prescription, specifically evaluating whether these prophylaxis interventions were initiated at the appropriate gestational age (prior to the 16th week). Patients who did not meet the clinical criteria for prophylaxis or those who declined to sign the Informed Consent Form were excluded.

Data were collected through structured questionnaires administered to the participants during their hospital stay or outpatient visit. The instruments evaluated sociodemographic profiles, medical and family history, and obstetric complications. In addition to the interviews, data collection involved a structured review of prenatal care records and electronic medical records. This review was conducted by trained researchers using a standardized data extraction form to retrospectively audit the gestational age at which aspirin and calcium were prescribed. Because the study design is strictly cross-sectional, data extraction focused entirely on evaluating provider adherence to screening guidelines and prescription adequacy up to the 16th week of gestation, without tracking subsequent clinical outcomes or the development of PE.

Crucially, the findings regarding calcium prescription must be interpreted within the specific clinical guideline context of the study period. Between July 2025 and January 2026, the active protocols issued by RBEHG and FEBRASGO still widely recommended universal calcium supplementation as a standard PE prophylaxis for all high-risk patients. Although these recommendations were subsequently withdrawn in early 2026 following emerging international evidence, provider practices evaluated in this study strictly reflected the clinical mandates and standard of care operating at the exact time of data collection.

The collected data were organized in Microsoft Excel and analyzed using Jamovi (version 2.3) and SPSS (version 21.0) softwares. Descriptive statistics were performed to characterize the sample using frequencies and percentages. To identify determinants of protocol adherence, the associations between independent factors and the dependent variables (adequate aspirin prescription and adequate calcium prescription) were analyzed. Bivariate logistic regression was performed to estimate crude Odds Ratios (OR) with their respective 95% confidence intervals (CI). Subsequently, multivariable logistic regression models were constructed using a standard forced-entry method based on clinical and epidemiological plausibility. The multivariable models included baseline major risk factors with adequate sample representation and the structural setting of initial care to calculate adjusted OR (AOR) while controlling for confounding. Critical risk factors with a small sample size and low prevalence were mathematically excluded from the regression models, because it would cause model instability. Multicollinearity among the independent variables in the multivariable models was formally assessed using tolerance values and the Variance Inflation Factor (VIF), with a VIF threshold of >5 used to identify significant collinearity. In the event of significant multicollinearity or severe clinical and statistical overlap between independent variables, separate multivariable models would be constructed for each main intervention to prevent model instability. A significance level of p<0.05 was adopted for all final models.

Following approval by the institutional ethics committee (CAAE: 87029724.4.0000.5149) 7.531.382.

## Results

A total of 100 patients at high risk for PE were eligible, including pregnant and postpartum women over 18 years of age who met the criteria for pharmacological PE prophylaxis and were included in the final analysis. The main demographic characteristics are summarized in table 1.

**Table 1:** Clinical and sociodemographic characteristics of pregnant women with risk factors for pre-eclampsia (n=100)

Variables		n(%)
Age		
	< 35 years	65(65)
	≥ 35 years	35(35)
Self-reported ethnicity		
	Black and "pardas" (brown)	84(84)
	White	12(12)
	Asian	2(2)
	Indigenous	2(2)
Follow-up location until at least the 16th week of gestation		
	At our tertiary center	38(38)
	At other centers	62(62)
Major risks for PE		
	Early pregnancy obesity (BMI ≥ 30 kg/m^2^)	60(60)
	History of PE *	23(36.5)*
	Chronic hypertension	42(42)
	Type 1 DM	4(4)
	Type 2 DM	14(14)
	Lupus	2(2)
	Multiple pregnancy	8(8)
	Pregnancy resulted from assisted reproduction	2(2)
	Chronic kidney disease	0(0)

* Nulliparous women (n=37) were excluded for this variable; Subtitle: DM = Diabetes Mellitus; PE = preeclampsia

Among the 100 patients included, 87 (87%) presented at least one major risk factor for PE. Notably, nearly half of the total sample (47%; n=47) exhibited an accumulation of multiple risk factors: 25% (n=25) had two major factors, 20% (n=20) had three, and 2% (n=2) presented four simultaneous major risk factors. When evaluating clinical overlaps within specific subgroups, we compared the prevalence of early pregnancy obesity between patients with and without metabolic comorbidities. We found that early pregnancy obesity significantly overlapped with CH, affecting 71.4% (30/42) of the hypertensive group compared to 51.7% (30/58) of non-hypertensive patients (p=0.047). Similarly, when comparing diabetic status, the prevalence of early obesity was significantly higher among patients with type 2 DM, reaching 92.8% (13/14) of cases compared to 54.6% (47/86) among those without type 2 DM (p=0.007). Additionally, a previous history of PE was observed in 28.6% (12/42) of patients with CH, although this specific comparison did not reach statistical significance (p=0.362).

The remaining 13 (13%) patients without major risks were classified as high risk due to the presence of at least two moderate factors. Within this subgroup, the most frequent characteristics were nulliparity (76.9%; n=10), followed by black or brown ethnicity (69.2%; n=9), maternal age ≥ 35 years (46.1%; n=6), and a positive family history of PE (46.1%; n=6). It is noteworthy that the majority of these women (69.2%; n=9) presented a combination of three or more of those moderate factors, reinforcing their high-risk status despite the absence of a major criteria. Regarding pharmacological prevention in this subgroup, only 23.1% (n=3) received adequate combined prophylaxis (aspirin and calcium). When analyzed independently, aspirin was prescribed appropriately in only 23.1% (n=3) of cases, while calcium supplementation reached 46.2% (n=6). Notably, 53.8% (n=7) of these patients did not receive any form of adequate prophylaxis, highlighting a significant failure in risk stratification for patients with multiple moderate factors.

Overall rates of adequate PE prophylaxis are shown in figure 1. Out of the 100 participants, 53.0% (n=53) received adequate combined prophylaxis (aspirin and calcium initiated before the 16th week). When analyzed independently, aspirin prophylaxis was adequate in 54.0% (n=54) of cases. Among the remaining 46 patients who did not receive adequate aspirin prophylaxis, 39.0% (n=39) were never prescribed the medication, and 7.0% (n=7) initiated it after the 16-week threshold. Regarding calcium, adequate prophylaxis was achieved in 77.0% (n=77) of the sample. In the group of 23 patients with inadequate calcium prophylaxis, 11.0% (n=11) were not prescribed the supplement at any point, and 12.0% (n=12) started the prophylaxis after the 16th week of gestation.

**Figure 1:**
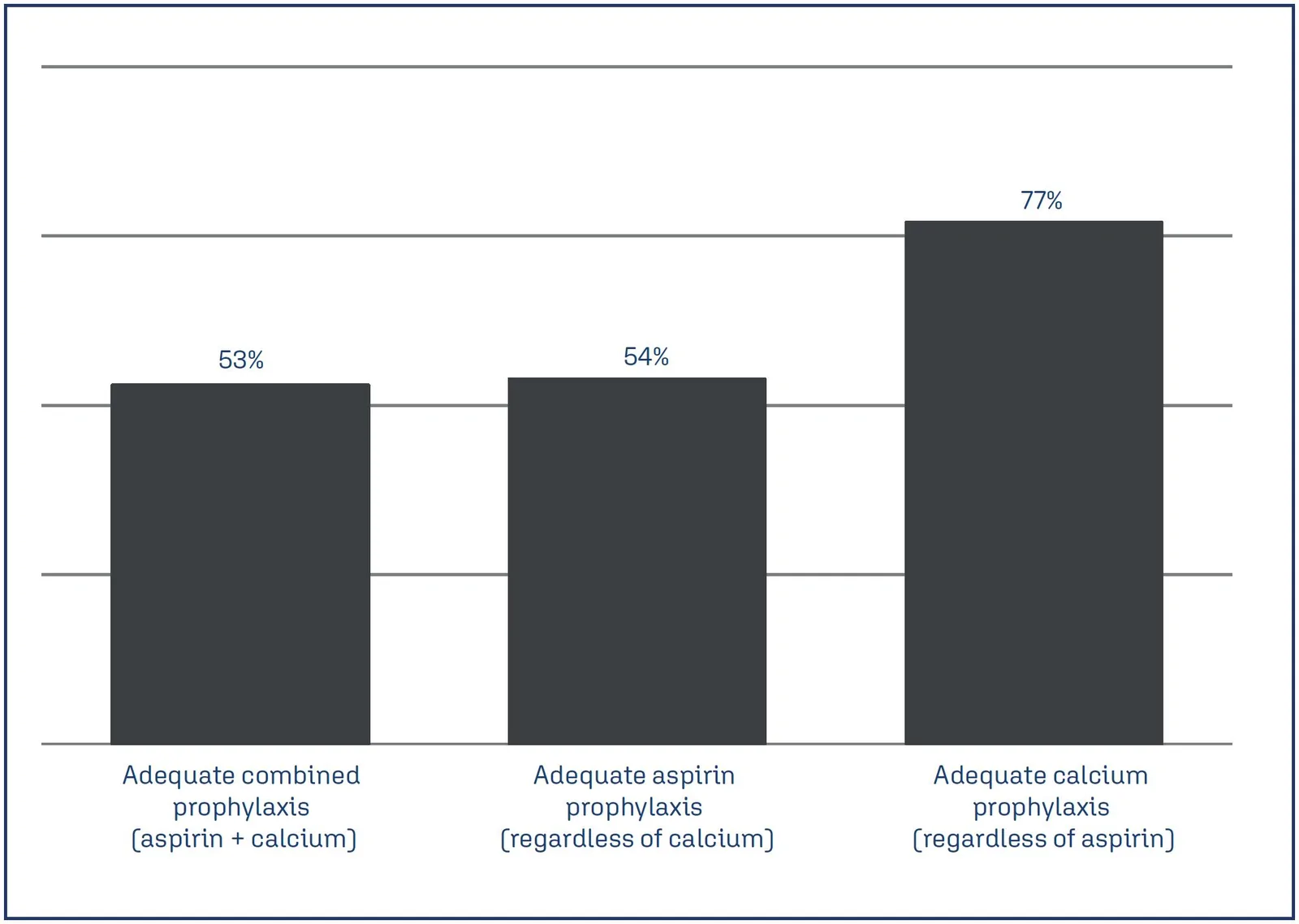
Adequacy of pre-eclampsia prophylaxis prescription in pregnant women at high risk for preeclampsia (n=100)

Bars represent the percentage of high-risk patients who received prescriptions according to clinical guidelines, defined as initiation before 16 weeks of gestation. Clinical and demographic characteristics of the patients who did not receive adequate prophylaxis are presented in table 2. Among the 46 patients with inadequate aspirin prophylaxis, a high prevalence of early pregnancy obesity (58.7%) and women aged 35 or more (34.8%) was observed. Similarly, for the 23 patients with inadequate calcium supplementation, 56.5% were obese and 39.1% were aged 35 years or older. Furthermore, among patients with inadequate calcium prophylaxis, an overwhelming rate of 95.7% also failed to receive adequate aspirin, indicating a systemic omission of the PE prophylaxis protocol in the referral network.

**Table 2:** Clinical and demographic characteristics of patients with inadequate prophylaxis for PE

Variables		Inadequate aspirin n=46 n(%)	Inadequate calcium n=23 n(%)
Age ≥ 35 years		16(34.8)	9(39.1)
Early pregnancy obesity		27(58.7)	13(56.5)
History of PE		9(19.6)	4(17.4)
Chronic hypertension		12(26.1)	4(17.4)
Type 2 DM		2(4.3)	0(0)
Multiple pregnancy		2(4.3)	1(4.3)
Prenatal care outside our center up to the 16th week		38(82.6)	19(82.6)
Cross-inadequacy			
	Also inadequate calcium	22(47.8)	—
	Also inadequate aspirin	—	22(95.7)

DM - Diabetes Mellitus; PE – preeclampsia

The associations between clinical and systemic factors and prophylaxis adequacy were further explored through regression models. Detailed results of the univariate and multivariate analyses for both aspirin and calcium are presented in table 3.

**Table 3:** Univariate and multivariate analysis of predictors for adequate aspirin and calcium prophylaxis for PE prevention (n=100)

Predictor	Aspirin OR (95% CI)	p-value	Aspirin AOR (95% CI)	p-value	Calcium OR (95% CI)	p-value	Calcium AOR (95% CI)	p-value
Chronic hypertension	3.54 (1.51–8.28)	0.004	3.07 (1.21–7.80)	0.018	4.63 (1.44–14.88)	0.010	4.11 (1.26–13.46)	0.019
Type 2 DM	6.29 (1.32–29.78)	0.021	5.06 (0.96–26.84)	0.056	—a	—a	—a	—a
Initial follow-up at our center*	5.94 (2.34–15.09)	<0.001	5.21 (1.95–13.91)	<0.001	3.76 (1.17–12.06)	0.026	3.24 (0.98–10.79)	0.054
Early pregnancy obesity	1.11 (0.50–2.47)	0.80	—	—	1.21 (0.47–3.10)	0.698	—	—
History of PE	1.58 (0.62–4.05)	0.33	—	—	1.67 (0.51–5.49)	0.401	—	—

AOR = adjusted Odds Ratio; CI = Confidence Interval; DM = Diabetes Mellitus; OR = Odds Ratio; PE = preeclampsia;

* Initial follow-up at our center: Patients whose prenatal care began at our institution until at least the 16th week of gestation.

a: Type 2 DM and calcium: Odds Ratio not calculable as all patients in this subgroup received the prescription (100% adequacy)

Univariate analysis of major risk factors revealed that CH and type 2 DM were among the strongest determinants for the correct prescription of both aspirin and calcium. For aspirin, CH (OR 3.54; 95% CI 1.51–8.28; p=0.004) and type 2 DM (OR 6.29; 95% CI 1.32–29.78; p=0.021) showed high clinical impact, while the setting of initial care emerged as a critical predictor, with patients followed at our tertiary center having significantly higher odds of adequate prophylaxis (OR 5.94; 95% CI 2.34–15.09; p<0.001). Conversely, early pregnancy obesity (OR 1.11; 95% CI 0.50–2.47; p=0.80) and a previous history of PE (OR 1.58; 95% CI 0.62–4.05; p=0.33) did not significantly influence aspirin prescription rates. Regarding calcium, CH (OR 4.63; 95% CI 1.44–14.88; p=0.010) and initial follow-up at our tertiary center (OR 3.76; 95% CI 1.17–12.06; p=0.026) significantly increased the odds of adequate prescription. Notably, 100% of patients with Type 2 DM received correct calcium prophylaxis, confirming its clinical relevance despite the mathematical inability to calculate an Odds Ratio for this subgroup. Similar to aspirin, early pregnancy obesity (OR 1.21; 95% CI 0.47–3.10; p=0.698) and history of PE (OR 1.67; 95% CI 0.51–5.49; p=0.401) remained non-significant for calcium.

Crucially, a massive cross-intervention overlap was observed, where 95.7% of patients with inadequate calcium prophylaxis also lacked adequate aspirin, indicating a single co-dependent clinical package at the bedside. To avoid extreme multicollinearity, these interventions were mutually excluded from each other's multivariable regression models. Other factors, such as lupus, antiphospholipid syndrome, and multiple pregnancy, were excluded from the models due to limited sample size, and while all patients with Type 1 DM (n=4) received adequate prophylaxis, 75% of them also had CH, limiting isolated conclusions. Finally, multicollinearity tests showed no excessive correlation between clinical factors and the setting of initial care (all VIF < 5).

In the final separate multivariable model for aspirin adequacy, initial care at our center remained a powerful independent predictor of prescription (AOR 5.21; 95% CI 1.95–13.91; p<0.001). CH also maintained an independent significant association with adequate prescription (AOR 3.07; 95% CI 1.21–7.80; p=0.018), while Type 2 DM showed a strong trend toward significance (AOR 5.06; 95% CI 0.96–26.84; p=0.056). Conversely, in the reciprocal multivariable model for calcium adequacy, CH emerged as the primary independent driver of protocol compliance (AOR 4.11; 95% CI 1.26–13.46; p=0.019). The setting of initial care at our tertiary center showed a strong independent trend but did not cross the significance threshold after adjustment (AOR 3.24; 95% CI 0.98–10.79; p=0.054). Early pregnancy obesity and history of PE remained non-significant in both adjusted models.

## Discussion

Our findings expose a profound, structured gap in guideline adherence within the prenatal referral network, characterized by an "all-or-nothing" prescription pattern. While combined pharmacological prophylaxis was achieved in only 53% of high-risk patients, separate multivariable analyses reveal distinct behavioral and systemic drivers for each drug when protecting the models against collinearity. On one hand, aspirin prescription was heavily dictated by the setting of initial care, with patients managed at our tertiary center over five times more likely to receive adequate therapy (AOR 5.21; 95% CI 1.95–13.91; p<0.001), exposing a severe structural barrier in peripheral screening. On the other hand, CH stood out as a robust independent driver for both adequate aspirin (AOR 3.07; 95% CI 1.21–7.80; p=0.018) and calcium prescription (AOR 4.11; 95% CI 1.26–13.46; p=0.019), indicating that prominent, classic maternal comorbidities successfully trigger provider adherence. Crucially, the massive cross-inadequacy overlap (95.7%) highlights that both interventions are virtually inseparable in clinical practice: once a provider overcomes clinical inertia to activate the protocol, the entire bundle is deployed, whereas missing the initial risk stratification leaves the patient entirely unprotected. Ultimately, this dichotomy underscores that the primary public health failure lies not in the mismanagement of individual therapies, but in a systemic failure of initial screening across lower-level healthcare facilities.

This systemic failure gains even greater relevance when examined in light of the profound shift in national and international guidelines regarding calcium supplementation. The definitive removal of routine calcium supplementation from the 2026 RBEHG/FEBRASGO protocols, driven by current landmark meta-analyses demonstrating a lack of robust preventive efficacy,^(9)^ fundamentally rewires the research priorities in PE prevention. Historically, universal calcium recommendations in Brazil were deployed as a broad public health measure, yet our findings expose that even when mandated as standard standard of care, its real-world implementation was severely flawed, leaving nearly a quarter of high-risk patients unprotected. Now that calcium has been clinically decoupled from PE prophylaxis, the spotlight must shift entirely toward optimizing low-dose aspirin protocols. Recent Brazilian data by Jardine and colleagues^(12)^ highlighted that even in specialized tertiary centers, non-adherence to prescribed PE prophylaxis among high-risk patients with CH reaches alarming rates of nearly 60% to 64%. However, as critically countered by Rezende and colleagues^(13)^, evaluating preventive efficacy becomes deeply flawed when structural barriers and screening failures prevent proper therapy initiation in the first place. The massive cross-inadequacy overlap observed in our study (95.7%) reinforces this perspective, proving that clinical neglect at the entry point of prenatal care acts as an absolute bottleneck. Therefore, future investigative efforts in Brazil must abandon the evaluation of obsolete dual-drug bundles and focus strictly on implementing early, reproducible aspirin screening.

The structural bottlenecks in screening and protocol implementation observed in our cohort are mirrored in global literature, which identifies significant knowledge and practice gaps among prenatal providers, especially in lower-level health facilities.^(14,15)^ Systematic reviews in low- and middle-income countries report alarmingly low rates of provider knowledge regarding aspirin prophylaxis, often below 20%.^(16)^ In light of implementation science frameworks, such as the Capability-Opportunity-Motivation-Behaviour (COM-B) model synthesized by Vinogradov and colleagues,^(17)^ the failure to achieve high prophylactic coverage is deeply rooted in deficits of psychological capability and physical opportunity within primary care settings. Healthcare systems frequently struggle with a translation gap where behavioral features, including provider unfamiliarity with timing thresholds and a lack of structured screening habits, actively prevent the clinical initiation of guidelines. Such evidence suggests that, even when national guidelines are available, the lack of specialized on-the-job training and poor protocol dissemination remain primary barriers to the effective implementation of PE prevention.^(14,15)^ Our results underscore the importance of standardized institutional protocols and specialized training in ensuring high adherence to prophylactic guidelines, guaranteeing that care remains consistent and based on international consensus regardless of where the patient initiates or continues their prenatal journey.

A particularly vulnerable subgroup identified in our study consists of patients whose high-risk status is defined by multiple moderate factors rather than a single major one. Only 21.3% of these individuals received adequate combined prophylaxis, a concerning gap that reflects a global trend where patients with only moderate risk factors are the least likely to receive aspirin, with rates ranging from 6% to 27%.^(18,19)^ In Brazil, this profile is increasingly relevant: while pregnancies at an advanced maternal age grow by 4.7% annually,^(20)^ black women continue to face a maternal mortality rate nearly twice as high as that of white women, a disparity that persists across all age groups and regions.^(21)^ Thus, our findings highlight a rising segment of the Brazilian population where chronological age intersects with deep-seated racial inequities. Clinicians must recognize that these factors, which are easily identified during a basic clinical examination at the onset of prenatal care, are critical determinants that can fundamentally alter gestational outcomes.

The multivariable analysis underscores a nuanced finding: while prominent clinical comorbidities like CH retain robust independent strength to drive protocol compliance (AOR 3.07 for aspirin and 4.11 for calcium), systemic factors such as institutional geography still play a critical role in aspirin coverage. This finding exposes a critical paradox in healthcare organizations. While robust global evidence demonstrates that community-based, decentralized antenatal care reduces health inequalities and improves perinatal outcomes when compared to hospital-centered models,^(22,23)^ our data show that the primary care network may act as a prophylactic bottleneck. Ensuring adequate prenatal care utilization is known to protect against adverse fetal outcomes,^(24)^ yet screening failures at lower-level facilities render the timely prescription of low-dose aspirin unacceptably dependent on specialized tertiary insertion. Behavioral models in implementation science suggest that this clinical inertia stems from a lack of structural screening habits at the entry point of care.^(17)^ Consequently, lower-level care may delegate risk management to specialized centers unless a glaring comorbidity like CH is present. To close this gap, health policies must implement automated, mandatory risk-stratification triggers across the entire referral network. In the obstetric setting, incorporating automated electronic health record reminders has been shown to drastically reverse clinical inertia, increasing adherence to preventive guidelines and optimizing early screening for at-risk pregnant women.^(25,26)^ Translating this digital tool into automated PE screening emerges as a pragmatic strategy to standardize care at the entry points of the prenatal network, thereby mitigating the institutional geographic bias observed here. However, technology alone cannot resolve systemic bottlenecks; recent implementation data suggest that future community-level interventions must prioritize health system strengthening, expanded workforce training, and broader clinical messaging.^(27)^ Expanding access to quality preventive care requires a primary health care model that brings services closer to where women live, leveraging continuous capacity building, structured coaching, and supervision for lower-level providers.^(28)^ Furthermore, establishing a shared care plan, which integrates targeted health-provider education with patient-centered advocacy, early behavioral modifications, and clear risk dissemination, is vital to ensure sustainable adherence to guidelines.^(29,30)^ Ultimately, a dual framework that synchronizes automated digital screening with robust professional development and community-level education is what will dismantle clinical inertia and successfully bridge the gap between primary and tertiary obstetric care.

Despite the valuable insights, our study has some limitations that must be acknowledged. First, the sample size (n=100) is relatively small and represents a convenience sample from a single tertiary center, which limits the generalizability of our findings to the entire Brazilian population or to different healthcare systems. The absence of a sample size calculation represents a methodological limitation that may affect the precision and reliability of our localized estimates for less prevalent risk profiles. This limited power also meant that rare but critical major risk factors, such as lupus, antiphospholipid syndrome, and multiple pregnancies, could not be adequately analyzed in our regression models. Furthermore, our sub-analysis regarding the subgroup of patients with only moderate risk factors comprised a very small number of cases (n=13), which represents a clear methodological limitation that precludes robust statistical inferences for this specific cohort. Nevertheless, from a clinical audit standpoint, this descriptive finding remains exploratory and highly illustrative, serving to point out a severe bedside vulnerability in routine risk stratification that warrants validation in larger multi-center studies. Furthermore, the cross-sectional and retrospective nature of the study relies on prenatal records and patient recall, which may be subject to information bias. Nevertheless, this design offers a pragmatic, real-world assessment of provider adherence to screening guidelines at a specific timeframe of prenatal care.

Nevertheless, this study has significant strengths. It offers a pragmatic assessment of the gap between clinical guidelines and routine bedside practice in a high-risk Brazilian cohort. By demonstrating that the setting of initial care overrides individual clinical risk profiles, we exposed a critical blind spot: the systematic neglect of standalone major risk factors, such as obesity and a previous history of PE, as well as cumulative moderate risk factors like nulliparity, advanced maternal age, and black or brown ethnicity. Our study uniquely highlights how structural disparities between tertiary and primary care dictate prophylaxis adequacy, offering a clear target for health policy interventions. These findings serve as a critical alert for the urgent need to standardize PE screening across the entire referral network, ensuring that high-risk patients are protected regardless of where they begin their prenatal care.

## Conclusion

Our findings suggest that pharmacological PE prophylaxis within the evaluated prenatal referral network is driven by a combination of high-visibility maternal risk factors, such as CH, and the structural setting of initial care. While prominent comorbidities successfully trigger protocol compliance, standalone or cumulative risk factors face structural bottlenecks at lower-level facilities, rendering optimal aspirin initiation heavily dependent on specialized tertiary insertion. Although recent national guidelines have officially withdrawn the recommendation for routine calcium supplementation due to a lack of robust preventive efficacy, our study evidences that even during the period when calcium was a mandatory component of standard protocols, its real-world implementation faced significant limitations. These observations indicate that the primary challenge within the local healthcare network may stem from early screening inertia rather than the specific drug combination recommended at the time. Although the limited sample size precludes broad generalizability, these results highlight the need for future multi-center studies to investigate logistical and provider-related barriers. Implementing clearer, standardized screening triggers at the entry points of prenatal care could potentially support providers and help prevent high-risk patients from being overlooked.

## Data Availability

The research data are described in the article presented.
